# Laser Excisional Biopsy of Bleeding Tumor near Newly Erupted Tooth in an 11-Month-Old Patient under General Anesthesia

**DOI:** 10.1155/2024/6668716

**Published:** 2024-01-18

**Authors:** Amir Mansour Shirani, Parnian Tadayonnezhad, Sarah Arzani, Seyed Omid Kiansadr, Nasser Kaviani

**Affiliations:** ^1^Oral Medicine Department, Dental School, Isfahan (Khorasgan) Branch, Islamic Azad University, Isfahan, Iran; ^2^Fellowship of Laser Therapy in Dentistry, RWTH Aachen University, Aachen, Germany; ^3^Dental School, Isfahan (Khorasgan) Branch, Islamic Azad University, Isfahan, Iran; ^4^Child Growth and Development Research Center, Research Institute for Primordial Prevention of Non-Communicable Disease, Isfahan University of Medical Sciences, Isfahan, Iran; ^5^Private Practitioner, and Researcher, Isfahan, Iran; ^6^Oral and Maxillofacial Department, Dental Research Center, Dental Research Institute, School of Dentistry, Isfahan University of Medical Sciences, Isfahan, Iran

## Abstract

Pyogenic granuloma (PG) is a well-known tumor-like growth that commonly appears in the gingiva of the oral cavity. This reactive lesion can develop in response to local irritation, chronic low-grade trauma, long-term low-grade infection, or hormonal imbalances. PG is more frequently observed in individuals during their second and third decades of life, although it can occur at any age. It is more prevalent in young females than males, but on rare occasions, it can be found in children, even infants. Several treatment options exist for PG lesions, which apply after a final diagnosis specified by biopsy and histopathological investigations. Surgical excision is the most common treatment choice for PG lesions. However, comparisons between laser therapy and surgical excision have shown numerous advantages for laser treatment, making it a preferred option for soft tissue lesions. Laser excision offers benefits such as precise and deep incisions, preservation of sterile conditions, improved hemostasis, avoidance of sutures, and less invasive procedures resulting in reduced intra- and postoperative discomfort, pain, and bleeding. This report is aimed at presenting a case of an 11-month-old infant with a gradually growing pyogenic granuloma (PG) lesion that appeared in the palate behind the upper left first deciduous tooth, with a duration of approximately 2 months. The parents reported a history of bleeding associated with the lesion, which emerged after the eruption of the mandibular tooth. An excisional biopsy was done by the application of an 810 nm diode laser under general anesthesia, and the specimen was evaluated by a pathologist. No posttreatment complications or relapses were observed in this case.

## 1. Introduction

Pyogenic granuloma (PG) is an exuberant, noncancerous lesion that occurs on the skin or in the oral cavity as an inflammatory hyperplasia [1, 2]. It was described for the first time by Hullihen in 1844, and later on, Hartzell introduced the term “pyogenic granuloma” or “granuloma pyogenicum” in 1904 [3, 4]. This reactive tumor-like lesion can appear in response to local irritation, chronic low-grade trauma, long-term low-grade infection, or hormonal imbalance [5]. The occurrence of PG is more frequent in the second and third decades of life, although it could be present at any age. It is more prevalent in young females than males, but on rare occasions, it can be found in children, especially infants [1, 6].

From a clinical point of view, PG is a painless, pedunculated, or sessile exophytic growth with a compressible and hemorrhagic smooth or lobulated surface. This mass can bleed easily, and its color depends on the vascularity level of the lesion [7, 8]. Marginal gingiva is the most commonly affected intraoral region, but it may also affect the tongue, palate, buccal mucosa, and lips. Furthermore, extraoral affected sites include the skin of the face, neck, upper and lower extremities, as well as the mucous membrane of the nose and eyelids [2, 8, 9].

There are several treatment options for PG lesions, which are applied after a final diagnosis specified by biopsy and histopathological investigations [9]. Conservative surgical removal of the lesion and elimination of the etiological factors has been introduced as a standard treatment option, as PG is not classified as a neoplastic lesion [10]. Various surgical modalities, including cryosurgery, cauterization with silver nitrate, sclerotherapy, injection of absolute ethanol, sodium tetradecyl sulfate and corticosteroids, as well as Nd:YAG, Er:YAG, diode, and CO2 lasers, have been presented [10, 11]. For example, laser excision of PG in a female case involving the dorsum of the tongue using a 940 nm diode laser resulted in a short healing period with no postoperative pain, ulceration, or scarring [11].

A simple comparison between the laser therapy and surgical excision approaches has shown many advantages for laser treatment, which has made it a preferred treatment option for soft tissue lesions. Surgical excision can have several complications, including intraoperative bleeding, developing more traumas, wound healing difficulties, and sterility preservation during the operation, which cannot be tolerable, especially for infants [12]. However, the main advantages of laser therapy include better performance of deep and precise incisions, the preservation of sterile conditions, better hemostasis, and less invasive procedures with less intra- and postoperative discomfort, pain, and bleeding [13–15].

According to the databases, there was no similar report of an infant with PG undergoing excisional biopsy by a diode laser while under general anesthesia. In this paper, the infant had a PG lesion that was bleeding, necessitating surgery to remove it. This report presents an 11-month-old infant case with a PG lesion and the application of a diode laser as a preferred treatment procedure under general anesthesia.

## 2. Case Report

**Figure 1:**
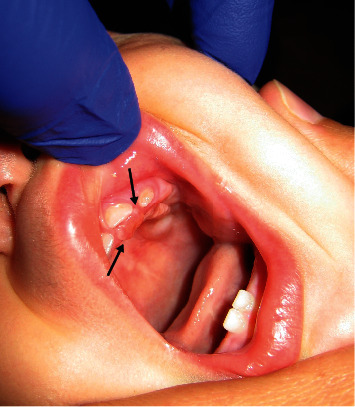
Tumor in the palatal side of the upper left first deciduous tooth.

An 11-month-old female infant presented with a 2-month history of a growing lesion in the palate behind the upper left first deciduous tooth, which appeared gradually. The lesion, according to the parents, had a history of bleeding and appeared after the eruption of the mandibular tooth. Clinical examination of this tumor-like growth demonstrated a single, sessile, and well-defined swelling on the palatal gingiva of the semierupted first incisor. It was red in color, soft to firm in consistency, and the size was approximately 1 cm (Figure 1). The radiographic examination did not reveal any kind of bone destruction.

**Figure 2:**
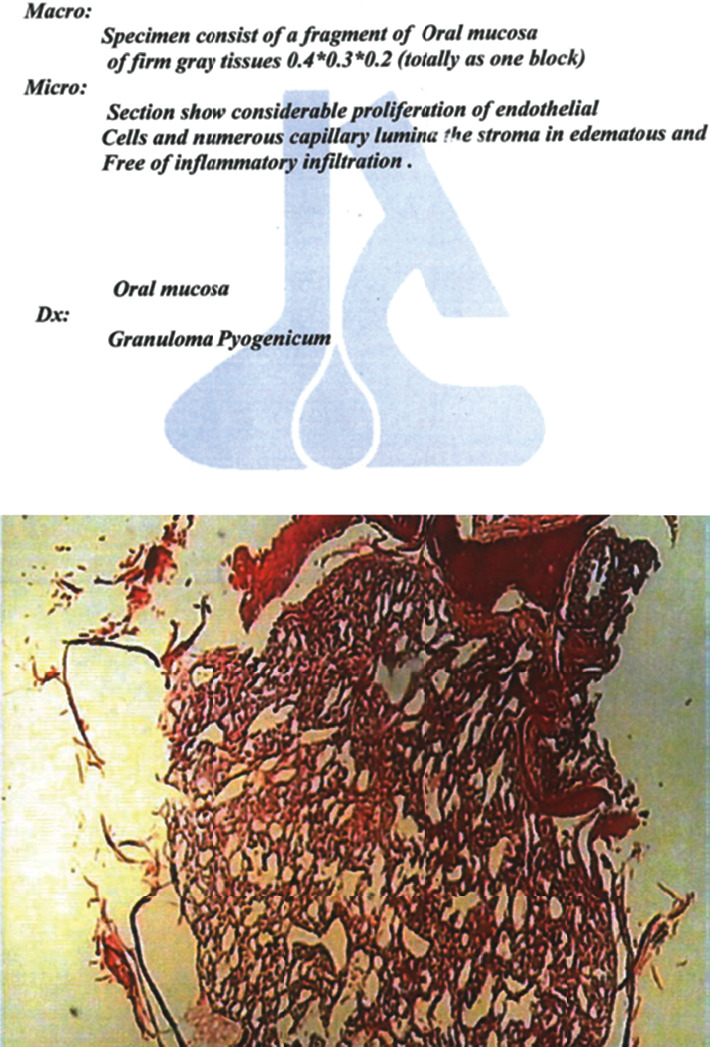
The histopathological result of the case.

Our clinical differential diagnosis included pyogenic granuloma, peripheral giant cell granuloma, and vascular malformations such as hemangioma. Differentiation between these lesions, aside from clinical appearance, requires a biopsy and histopathological assessment of the obtained biopsy (Figure 2). The final diagnosis was pyogenic granuloma based on the clinical and histopathological evidence. The cause of the lesion might be related to the trauma of the lower anterior deciduous tooth eruption, or it may have happened during feeding. A treatment plan was developed, which involved excisional biopsy and bleeding control utilizing diode laser under general anesthesia. Induction of anesthesia was performed with thiopental Na 5 mg/kg, fentanyl 2 *μ*g/kg, and atracurium 0/6 mg/kg. After nasotracheal intubation with an appropriate nasotracheal tube, anesthesia was continued with O_2_/N_2_O and isoflurane. At the end of surgery, muscle relaxants were reversed with neostigmine and atropine, and the child was monitored in the recovery room [16].

**Figure 3:**
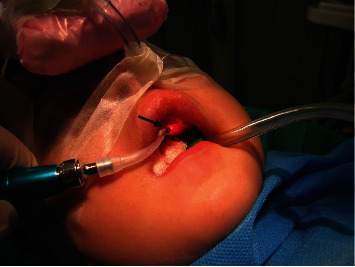
Tumor removal with a diode laser at 810 nm under general anesthesia.

**Figure 4:**
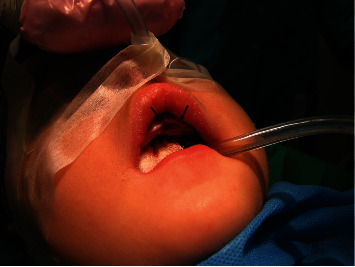
No bleeding after tumor removal.

**Figure 5:**
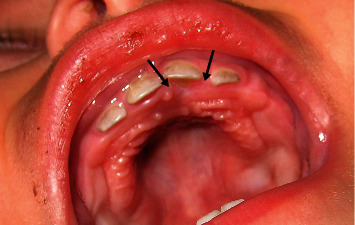
No relapse of the tumor in a 1-month follow-up visit.

After applying general anesthesia, a diode laser with an 810 nm wavelength (Gigaa Laser CHEESE, China) was utilized in continuous wave mode. The laser operated at a power output of 2.5 W and was employed in contact mode with a 400 *μ*m fiber, positioned perpendicularly for the complete excision of the lesion (Figures 3 and 4). The histopathological assessment of the biopsy specimen confirmed the diagnosis of pyogenic granuloma. Postoperative instructions were given to the parents for good oral hygiene to keep the area clean, and the patient was discharged. The 1-month follow-up visit showed complete healing of the surgical side with no signs of recurrent lesions (Figure 5). The patient did not refer to the office over the next few years after surgery which shows no relapse of the lesion.

## 3. Discussion

Oral PG is a vascular, benign tumor-like growth that could occur at any age, but it is more frequent in young adults and women [17, 18]. It is generally accepted that PG is formed in response to different stimulations like traumatic injuries, certain medications, and recurrent local irritations. Other factors that have been linked to the development of this lesion include abnormal tooth development, primary tooth injury, and, as in this case, tooth eruption, which causes chronic trauma to the gums [14, 19–21].

In this study, a lesion was developed in the palate behind the upper left first deciduous tooth due to the eruption of lower anterior deciduous teeth. While gingiva is the most common site for PG, the growth in the present case occurred on the hard palate behind the first deciduous tooth, which is a rare place for its occurrence [22]. However, bleeding may occur in response to minor trauma; as has been noted in this case's history, representing pain or any other symptoms is not probable [22]. Oral PG clinically manifests as an exophytic, small, red erythematous lesion on a pedunculated or, as in this case, a sessile base [23].

Surgical excision is the most common treatment modality for PG, but different forms of lasers have been used because of their advantages. Different types of lesions, such as fibrotic lesions, gingival growths, and mucoceles, can be safely and quickly removed by lasers [24]. In this regard, Asundaria and Tavargeri claimed that laser treatment reduced bleeding and postoperative pain in a pediatric patient with traumatic fibroma of the tongue while also promoting rapid healing and little scarring [25]. A case of mucocele that was treated with a diode laser was presented by Saha et al. According to the authors, using a laser to treat mucoceles in pediatric children is an efficient, quick, uncomplicated, bloodless, and generally acceptable method [26].

The benefits of laser therapy include reduced bleeding and bacteremia, instant sterilization, less need for sutures and/or dressings postsurgery, a substantial reduction in pain and edema during and after surgery, a more effective healing process, and higher patient satisfaction [24]. Moreover, it is a less invasive procedure in regions where aesthetics is essential compared to scalpel and cryosurgery treatments [22].

In a case reported by Powell et al., which was one of the first uses of Nd:YAG laser for PG excision, superior coagulation characteristics as well as a decrease in bleeding were reported compared to other methods [27]. Fekrazad et al., by applying Er:YAG laser for excision of PG, declared that CO_2_ and Er:YAG lasers are more adequate for cutting than Nd:YAG and diode lasers, considering their higher absorption in water, less penetration, and coagulation [13].

Diode lasers, as they are manufactured by semiconductor crystals of aluminum (800 nm) or indium (900 nm), gallium, and arsenide, penetrate deeply into the mucosa, but they are hardly absorbed by the dental hard tissue at the particular wavelengths mentioned [14]. Rai et al. used an 808 nm diode laser as an effective option for the treatment of an intraoral PG. The authors recommended laser therapy because it is less invasive, limits hemorrhage during surgery, is easy to use, and has a low recurrence rate [8]. Relatively, Asnaashari et al., after the application of a diode laser to a pediatric patient with an oral PG lesion, expressed minimal discomfort inter- and postoperation [21]. In this regard, the diode laser is more suitable than other types of lasers, assuming a smaller size of lesion and a lower cost [20].

The treatment plan for the present case involved an excisional biopsy and bleeding control using a diode laser under general anesthesia. The decision to induce anesthesia was primarily based on the young age of the child. However, it is worth noting that similar case reports have successfully treated pyogenic granuloma (PG) using a diode laser with local anesthesia. For instance, Khan and Jindal reported a case of an 11-year-old boy with a PG in the anterior hard palatal region, adjacent to the marginal gingiva of the central incisors. They successfully treated the case using a diode laser with a wavelength of 808 ± 10 nm, while the procedure was performed with topical lidocaine anesthetic spray for anesthesia [28]. Similarly, Pisano et al. described a case of a swelling PG in the lower lip of an 11-year-old female patient. In their report, the lesion was excised successfully using a 980 nm diode laser in continuous wave mode after establishing anesthesia through the infiltration of 4% articaine with epinephrine 1 : 200,000. This conservative approach with the diode laser proved to be a nonstressful method for the pediatric patient [29].

Many other researchers have recently preferred diode lasers for PG excision [15, 30, 31]. In this current study, a diode laser at the wavelength of 810 nm for an infant undergoing general anesthesia was used for the first time, with no complication.

## 4. Conclusion

Oral pyogenic granuloma (PG) is a reactive lesion that is relatively uncommon in children, particularly infants. Laser removal provides a safe and effective approach due to several advantages it offers, including reduced bleeding, faster healing, improved patient comfort, and a lower recurrence rate. Therefore, laser treatment has many advantages as the gold standard due to its numerous advantages. In line with this, the treatment plan for the present case involved performing an excisional biopsy and bleeding control utilizing a diode laser under general anesthesia. This intervention was specifically designed for an infant who had been diagnosed with PG.

## Data Availability

The corresponding author will provide any data in support of this case regarding the patient's confidentiality upon reasonable request.
